# Clinical usefulness of hybrid endoscopic submucosal dissection for T1b colorectal carcinomas ≤20 mm to ensure adequate vertical margins

**DOI:** 10.1002/deo2.70030

**Published:** 2024-10-24

**Authors:** Yudai Takehara, Ken Yamashita, Shin Morimoto, Fumiaki Tanino, Noriko Yamamoto, Yuki Kamigaichi, Hidenori Tanaka, Hidehiko Takigawa, Yuji Urabe, Toshio Kuwai, Koji Arihiro, Shiro Oka

**Affiliations:** ^1^ Department of Gastroenterology Hiroshima University Hospital Hiroshima Japan; ^2^ Gastrointestinal Endoscopy and Medicine Hiroshima University Hospital Hiroshima Japan; ^3^ Department of Anatomical Pathology Hiroshima University Hospital Hiroshima Japan

**Keywords:** colorectal carcinoma, endoscopic mucosal resection, endoscopic submucosal dissection, recurrence, tumor

## Abstract

**Objective:**

To evaluate endoscopic resection strategies for cT1b colorectal carcinomas (CRCs) ≤20 mm to determine strategies that enable adequate vertical margins (VMs).

**Methods:**

We enrolled 128 consecutive patients with cT1b colorectal carcinomas ≤20 mm resected by endoscopic mucosal resection or hybrid endoscopic submucosal dissection (ESD). Tumor lifting conditions after submucosal injection were classified into type A (lifting, soft dome‐like), type B (lifting, hard trapezoid‐like), and non‐lifting (positive non‐lifting sign). Predictors of positive VMs (VM 1) and adequate VMs were identified.

**Results:**

All non‐lifting tumors were resected by hybrid ESD and VMs were ≥500 µm. Vertical margin 1 tumors were only found in the endoscopic mucosal resection group, in which, the proportion of type B tumors with VM 1 was significantly higher than that of tumors with negative VMs (*p* < 0.01). Type A tumors showed no significant between‐group differences. Among type B tumors, the proportion of VMs ≥500 µm was significantly higher (*p* < 0.01) and the VM distance was significantly longer (*p* < 0.01) in the hybrid ESD group than in the endoscopic mucosal resection group.

**Conclusions:**

Hybrid ESD can be selected for type B tumors to ensure adequate VMs.

## INTRODUCTION

The number of treated submucosal invasive (T1) colorectal carcinomas (CRCs) and endoscopic resections (ERs) for T1 CRCs have been increasing annually.^1^ The Japanese Society for Cancer of the Colon and Rectum (JSCCR) 2022 guidelines^2^ state that intramucosal and superficial submucosal invasive (T1a) CRCs (invasion depth <1000 µm) are indications for en bloc ER; moreover, intestinal resection with lymph node dissection is indicated for deep submucosal invasion (T1b) CRCs (≥1000 µm). However, pathological conditions of T1b CRCs with an extremely low lymph node metastasis risk are identified^3^, ^4^, ^5^, ^6^; therefore, indications for ER are being considered. A negative vertical margin (VM) is an absolute requirement for curative ER according to the JSCCR, National Comprehensive Cancer Network, and European Society for Medical Oncology guidelines.^2^, ^7^, ^8^ The current Japanese Classification of Colorectal, Appendiceal, and Anal Carcinoma states that desirable resection by ER (VM distance) is ≥500 µm from the deepest invasion front of the carcinoma to the marginal termination resected.^9^ Complete en bloc resection with VM distance ≥500 µm during ER for T1 CRC reduces the metastatic recurrence risk after additional surgery.^10^ An adequate VM distance is required; however, ER strategies for T1 CRC that ensure adequate VMs remain unclear.

Endoscopic mucosal resection (EMR), the primary standard method for colorectal tumors,^11^, ^12^, ^13^ is limited to lesions ≤20 mm because of the snare diameter. When en bloc resection is possible, EMR is a standard method for non‐pedunculated tumors ≤20 mm suspected to be cancer.^14^, ^15^ Submucosal injection is important for EMR, and EMR is difficult when lesions display positive non‐lifting signs,^16^, ^17^ which are commonly seen in tumors with fibrosis or cT1b CRCs. In contrast, endoscopic submucosal dissection (ESD) enables complete en bloc resection irrespective of lesion size^18^, ^19^, ^20^ and can be performed for lesions ≤20 mm with submucosal fibrosis or cT1b CRCs that are difficult to resect using en bloc EMR. ESD is technically more difficult than EMR requires more time and poses a higher perforation risk.^18^, ^20^ Recently, hybrid ESD, a modified ESD technique, has gained popularity.^21^, ^22^, ^23^, ^24^, ^25^ It simplifies submucosal dissection, resulting in decreased procedure times and complications. We retrospectively evaluated ER strategies for cT1b CRCs ≤20 mm resected by EMR or hybrid ESD based on the findings following submucosal injection to determine strategies that ensure adequate VMs.

## METHODS

### Patients

This retrospective, single‐center study included 128 consecutive patients with 128 cT1b CRCs ≤20 mm resected by EMR or hybrid ESD at Hiroshima University Hospital between August 2009 and December 2022. For cT1b CRCs ≤20 mm, 108 patients underwent primary surgery and 50 underwent conventional ESD during the same period; these patients were excluded. Four pedunculated tumors were excluded. We focused on EMR and performed comparisons in terms of snaring. Tumors (*n* = 124) resected by EMR (*n* = 93) or hybrid ESD (*n* = 31) were included. After explaining the procedure to the patients, ER was performed in older patients or patients with serious complications, those who refused surgical resection and preferred ESD, and those with Rb lesions (from peritoneal reflection to the upper border of the puborectal muscle attachment) that could diminish their quality of life postoperatively because of possible colostomy and defecation, urination, and sexual dysfunctions.

**FIGURE 1:**
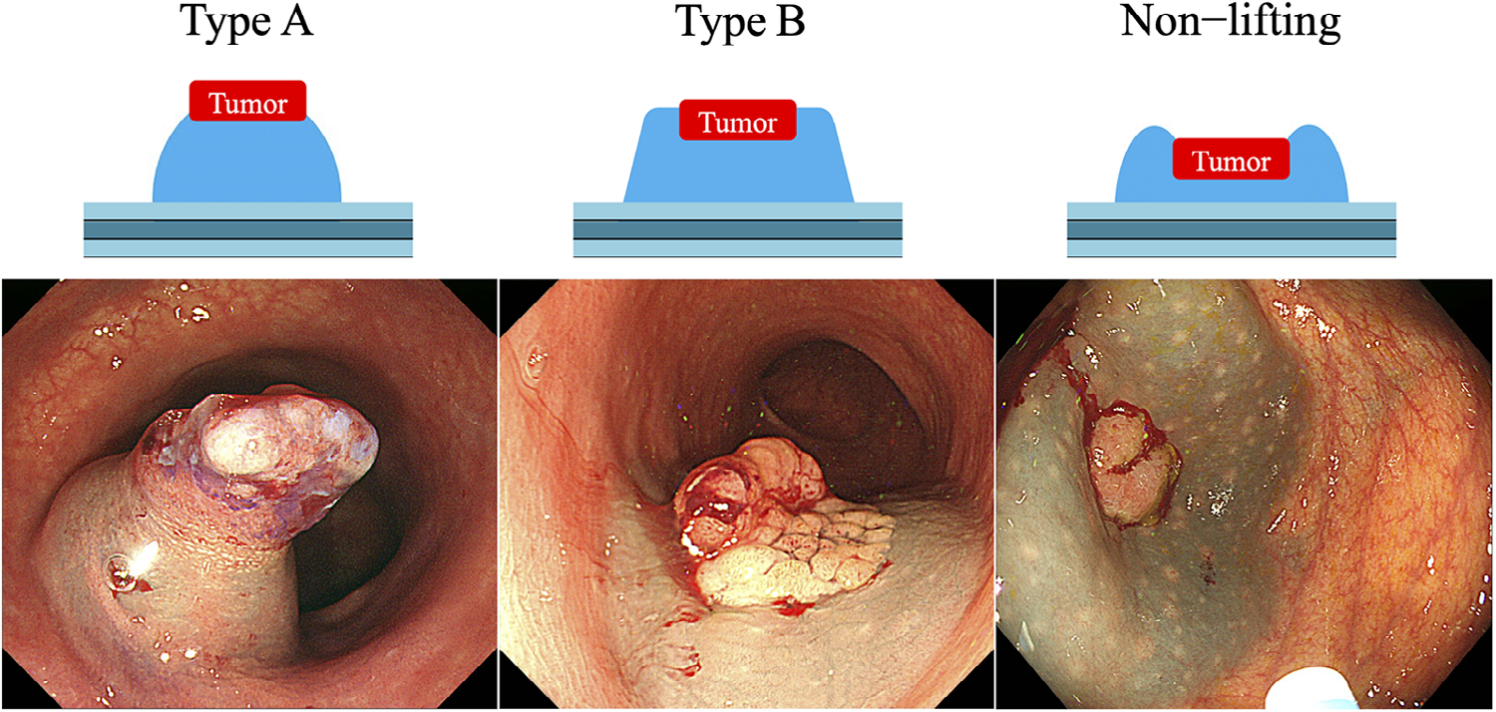
Classifications of tumor lifting conditions after submucosal injection: type A, complete lifting and soft dome‐like form; type B, complete lifting and hard trapezoid‐like form; and non‐lifting, slight lifting with better lifting of the surrounding mucosa or no lifting.

Tumor lifting conditions after submucosal injection were classified before ER into type A (complete lifting with a soft, dome‐like form), type B (complete lifting with a hard, trapezoid‐like form), and non‐lifting (positive non‐lifting sign^16^, ^17^ that indicated slight lifting with better lifting of the surrounding mucosa or no lifting; Figure 1). Two expert endoscopists retrospectively assessed the lifting conditions by reviewing images after submucosal injection and determined the lifting types before reviewing the ER procedure results. Interobserver variability for the classification was <5%.

### EMR and hybrid ESD procedures

Five highly experienced endoscopists experienced in >30 colorectal ESD procedures performed ERs and selected the methods for cT1b CRCs based on the clinicopathological lesion characteristics.

During EMR, a 10% glycerin solution containing indigo carmine (indigo carmine/solution: 0.2/20 mL) was injected into the tumor's submucosal layer. The elevated lesion was excised by constriction with a 10‐, 15‐, or 20‐mm snare (SnareMaster; Olympus or Captivator; Boston Scientific).

During hybrid ESD, a local solution with the same composition as that used for EMR was injected into the tumor's submucosal layer. DualKnife (Olympus) or 15‐mm SOUTEN (Kaneka Medics) was used as the ESD knife. A 20‐mm SnareMaster snare (Olympus) or 15‐mm SOUTEN (Kaneka Medics) was used for snaring. The SM dissection extent before snare resection was determined by each endoscopist depending on the lesion and situation. A transparent tip hood made submucosal layer entry easier and ensured good visibility.^26^ All lesions were diagnosed as cT1b CRCs; we dissected the submucosa just above the muscle layer and below the tumor.

To form an ideal tumor lifting condition that facilitates resection, the lesion was positioned towards the 6 o'clock direction on the screen, and the solution was injected from the lesion's anal side using a down‐angle to ensure adequate injection of the solution directly beneath it. The submucosal injection was basically performed as a single injection to prevent solution spread. The electrosurgical unit was VIO300D (ERBE). During EMR, the ENDO CUT Q mode (effect2, time2, duration3) was used for snaring. During hybrid ESD, the ENDO CUT I (effect3, time2, duration2), swift coagulation (effect3, 30 W), and ENDO CUT Q (effect2, time2, duration3) modes were used for mucosal incision, SM dissection, and snaring, respectively.

### cT1b CRC definition

We performed normal endoscopic observation, magnified endoscopy of the pit pattern using magnifying chromoendoscopy, endoscopic ultrasonography (EUS), and barium enema evaluations for cT1b CRC diagnosis. Expanded appearance, deeply depressed surfaces, folds converging toward the tumor, type V_I_‐severe^27^ or V_N_ pit pattern on chromoendoscopy, submucosal layer invasion on EUS, and a smooth surface, fold convergency, or eccentric rigidity in barium enema led to suspicion of cT1b CRC.^28^ After considering all factors, cT1b CRC was diagnosed.

### Histopathological diagnosis

Endoscopically resected specimens were fixed with 10% formalin and cut into 2‐mm‐thick sections. Pathological characteristics (tumor diameter, histologic type, submucosal invasion depth, lymphovascular invasion, budding grade, and resection margin) were evaluated according to the JSCCR 2022 guidelines.^2^ En bloc resection was defined as single‐piece resection. VM1 was defined as the presence of tumor cells at the submucosal VM of resected specimens. The distance from the tumor invasive front to the VM of resected specimens (VM distance) was measured using a digital measurement system (DP2‐SAL; Olympus). VM distance in high‐grade dysplasia was defined as the distance from the muscularis mucosae to the VM of resected specimens. VM distance of VM1 tumors was defined as 0 µm. According to the treatment strategy after ER for pT1 CRCs indicated by the JSCCR 2022 guidelines,^2^ we strongly recommended intestinal resection with lymph node dissection for VM1 lesions. For VM‐negative pT1b CRCs, resection with intestinal lymph node dissection was considered; therefore, we consulted with surgeons and selected a treatment policy based on histopathological risk factors (main histology, vascular invasion, and budding grade), patients’ wishes, and background factors. One expert gastrointestinal pathologist, blinded to the ER method, diagnosed and measured vertical tumor margin distances.

### Evaluation

EMR and hybrid ESD groups were analyzed to identify predictors of VM1 and adequate VM distance, including location, tumor size, gross type, tumor lifting conditions, histologic findings (main histology, histology at the deepest invasive front, submucosal invasion depth, vascular invasion, and budding grade), rebleeding, and perforation.

### Statistical analysis

All variables are expressed as mean with standard deviation, median with range, or number with percentage. Distributions of continuous variables were compared using the Student's *t*‐test or Mann–Whitney *U* test. Associations between categorical variables and outcomes were examined using Pearson's chi‐square test and Fisher's exact test. Statistical analyses were performed using JMP software version 17.0.0 (SAS Institute Inc.), with statistical significance set at *p* < 0.05.

## RESULTS

### Comparison of overall outcomes

Table 1 shows the clinicopathological characteristics and outcomes of tumors in the EMR (93 tumors) and hybrid ESD (31 tumors) groups. The mean age and sex ratio (male:female) in the EMR and hybrid ESD groups were 70.0 ± 9.0 years and 63:30 and 69.8 ± 11.3 years and 18:13, respectively. The proportions of rectal tumors (32.3% [10/31] vs. 12.9% [12/93], *p* < 0.05), type B tumor lifting condition (32.3% [10/31] vs. 24.7% [23/93], *p* < 0.01), budding grade 2/3 (45.2% [14/31] vs. 23.7% [22/93], *p* < 0.05), and VM ≥500 µm (90.3% [28/31] vs. 62.4% [58/93], *p* < 0.01) were significantly higher in the hybrid ESD group than in the EMR group. The proportion of VM1 tumors was significantly higher in the EMR group (12.9% [12/93] vs. 0% [0/31], *p* < 0.05). Other clinicopathological characteristics and outcomes showed no significant between‐group differences. All three non‐lifting tumors were resected using hybrid ESD. For two of them, EMR was converted to hybrid ESD on the same day because of tumor lifting conditions. For three tumors, a certain distance between the deepest invasive tumor front and muscle layer was confirmed preoperatively using EUS; therefore, en bloc resection using hybrid ESD was considered possible. The VM distance of these non‐lifting tumors was ≥500 µm. No tumor required conversion from ESD to EMR. Sixty‐five patients with 65 tumors underwent additional surgery after ER, with no local tumor remnants. The mean time for hybrid ESD was 29.9 ± 14.6 min, and clip closure to the post‐ER ulcer was generally not performed after the procedure. Exceptionally, clipping only at the perforation or damaged site was performed in cases of perforation or severe damage to the muscle layer at the post‐ER ulcer.

**TABLE 1:** Clinicopathological characteristics and outcomes of tumors in the endoscopic mucosal resection (EMR) and hybrid endoscopic submucosal dissection (ESD) groups.

	EMR group	Hybrid ESD group	
Variables	*n* = 93	*n* = 31	*p*‐value
Age, years, mean ± SD	70.0 ± 9.0	69.8 ± 11.3	0.9184
Sex, *n* (%)			
Male	63 (67.7)	18 (58.1)	0.3852
Female	30 (32.3)	13 (41.9)	
Location, *n* (%)			
Colon	81 (87.1)	21 (67.7)	0.0270
Rectum	12 (12.9)	10 (32.3)	
Tumor size, mm, mean ± SD	11.8 ± 5.0	13.8 ± 4.3	0.0446
Gross type, *n* (%)			
Protruded	75 (80.7)	17 (54.8)	0.0082
Superficial	18 (19.3)	14 (45.2)	
Tumor lifting condition, *n* (%)			
Type A	70 (75.3)	18 (58.1)	0.0053
Type B	23 (24.7)	10 (32.3)	
Non‐lifting	0 (0)	3 (9.6)	
Main histology, *n* (%)			
tub/pap	91 (97.9)	30 (96.7)	1.0000
por/sig/muc	2 (2.1)	1 (3.2)	
Histology at the deepest invasive front, *n* (%)			
tub/pap	82 (88.2)	28 (90.3)	1.0000
por/sig/muc	11 (11.8)	3 (9.7)	
Histologic findings, *n* (%)			
HGD	10 (10.8)	2 (6.5)	0.3067
T1a carcinoma	5 (5.4)	0 (0)	
T1b carcinoma	78 (83.8)	29 (93.5)	
SM depth, µm, median [range]	2000 [1200–3500]	2000 [1300–3000]	0.9838
Vascular invasion positive, *n* (%)	27 (29.0)	14 (45.2)	0.1236
Budding grade 2/3, *n* (%)	22 (23.7)	14 (45.2)	0.0381
En bloc resection, *n* (%)	91 (97.9)	31 (100)	1.0000
VM distance, µm, median [range]	750 [325–1600]	700 [500–1800]	0.3660
VM ≥500µm, *n* (%)	58 (62.4)	28 (90.3)	0.0032
VM1, *n* (%)	12 (12.9)	0 (0)	0.0364
Rebleeding, *n* (%)	2 (2.2)	1 (3.2)	1.0000
Perforation, *n* (%)	0 (0)	0 (0)	–

Abbreviations: EMR, endoscopic mucosal resection; ESD, endoscopic submucosal dissection; HGD, high‐grade dysplasia; muc, mucinous adenocarcinoma; pap, papillary adenocarcinoma; por, poorly differentiated adenocarcinoma; SD, standard deviation; sig, signet‐ring cell carcinoma; SM, submucosal; tub, tubular adenocarcinoma; VM, vertical margin.

### Risk factors for VM1 and VM <500 µm

VM1 tumors were only seen in the EMR group; therefore, we compared the clinical characteristics of VM1 and VM‐negative tumors in the EMR group to identify clinical factors associated with VM1 (Table 2). The proportion of type B lifting condition was significantly higher for VM1 tumors than for VM‐negative tumors (100% [12/12] vs. 13.6% [11/81], *p* < 0.01). No significant between‐group differences were observed in location, tumor size, or gross type of VM1 and VM‐negative tumors. Table 3 presents univariate and multivariate logistic regression analyses of tumor features associated with VM <500 µm (colon location, tumor size ≤15 mm, superficial type, type B lifting condition, and pathological T1b); the results showed that the type B lifting condition was significantly associated with VM <500 µm.

**TABLE 2:** Clinical characteristics of tumors in the vertical margin 1 (VM1) and VM‐negative endoscopic mucosal resection (EMR) groups.

	VM1	VM‐negative	
Variables	*n* = 12	*n* = 81	*p*‐value
Age, years, mean ± SD	69.4 ± 13.4	70.1 ± 8.2	0.4717
Sex, *n* (%)			
Male	7 (58.3)	56 (69.1)	0.5146
Female	5 (41.7)	25 (30.9)	
Location, *n* (%)			
Colon	10 (83.3)	71 (87.7)	0.6508
Rectum	2 (16.7)	10 (12.3)	
Tumor size, mm, mean ± SD	11.3 ± 4.8	11.9 ± 5.1	0.6894
Gross type			
Protruded	10 (83.3)	65 (80.3)	1.0000
Superficial	2 (16.7)	16 (19.7)	
Tumor lifting condition, *n* (%)			
Type A	0 (0)	70 (86.4)	<0.0001
Type B	12 (100)	11 (13.6)	
Histologic findings, *n* (%)			
HGD	1 (8.3)	9 (11.1)	0.6327
T1a carcinoma	0 (0)	5 (6.2)	
T1b carcinoma	11 (91.7)	67 (82.7)	
SM depth, µm, median [range]	2800 [2275–3000]	2000 [1200–3600]	0.1443

Abbreviations: EMR, endoscopic mucosal resection; ESD, endoscopic submucosal dissection; muc, mucinous adenocarcinoma; pap, papillary adenocarcinoma; por, poorly differentiated adenocarcinoma; SD, standard deviation; sig, signet‐ring cell carcinoma; tub, tubular adenocarcinoma; VM, vertical margin.

**TABLE 3:** Univariate and multivariate analyses of factors independently predictive of vertical margin (VM) <500 µm tumors.

	Univariate analysis		Multivariate analysis	
Characteristics	OR (95% CI)	*p*‐value	OR (95% CI)	*p*‐value
Colon location	1.58 (0.56–5.15)	0.3999		
Tumor size ≤15 mm	2.52 (0.86–9.23)	0.0931	3.20 (0.93–13.76)	0.066
Superficial type	2.18 (0.91–5.18)	0.0798	1.67 (0.61–4.48)	0.3135
Type B lifting condition	9.00 (3.74–23.01)	<0.0001	9.09 (3.65–24.31)	<0.0001
Pathological T1b	1.08 (0.39–3.29)	0.8818		

Abbreviations: CI, confidence interval; OR, odds ratio; SM, submucosal; VM, vertical margin.

### Risk factors for short VMs according to tumor lifting conditions

The results revealed the tumor lifting condition was likely associated with the VM distance; therefore, we compared the clinicopathological characteristics of type A and type B tumors between the EMR and hybrid ESD groups to determine whether the VM distance was different (Tables 4 and 5). Type A tumors exhibited no significant between‐group differences in clinicopathological characteristics or outcomes, including the VM distance (1000 [652–2025] mm vs. 900 [500–1800] mm, *p* = 0.54) and proportion of VMs ≥500 µm (80.0% [56/70] vs. 88.9% [16/18], *p* = 0.51). For type B tumors, the hybrid ESD group showed significantly longer VM distance (700 [500–1925] mm vs. 0 [0–400] mm, *p* < 0.01) and higher proportion of VMs ≥500 µm (90.0% [9/10] vs. 8.7% [2/23], *p* < 0.01) than did the EMR group. We also compared the clinical characteristics of tumors with type A and B lifting conditions to identify any pre‐injection factors that could predict type B lifting conditions (Table 6). No significant between‐group differences were observed in the location, tumor size, and gross type.

**TABLE 4:** Clinicopathological characteristics and outcomes of tumors with the type A lifting condition in the endoscopic mucosal resection (EMR) and hybrid endoscopic submucosal dissection groups.

	EMR group	Hybrid ESD group	
Variables	*n* = 70	*n* = 18	*p*‐value
Age, years, mean ± SD	70.1 ± 8.5	66.8 ± 11.1	0.1790
Sex, *n* (%)			
Male	48 (68.6)	11 (61.1)	0.5814
Female	22 (31.4)	7 (38.9)	
Location, *n* (%)			
Colon	62 (88.6)	13 (72.2)	0.1294
Rectum	8 (11.4)	5 (27.8)	
Tumor size, mm, mean ± SD	12.1 ± 5.2	12.6 ± 4.0	0.7221
Gross type, *n* (%)			
Protruded	59 (84.3)	12 (66.7)	0.1044
Superficial	11 (15.7)	6 (33.3)	
Main histology, *n* (%)			
tub/pap	70 (100)	17 (94.4)	0.2045
por/sig/muc	0 (0)	1 (5.6)	
Histology at the deepest invasive front, *n* (%)			
tub/pap	61 (87.1)	17 (94.4)	0.6800
por/sig/muc	9 (12.9)	1 (5.6)	
Histologic findings, *n* (%)			
HGD	9 (12.9)	0 (0)	0.1407
T1a carcinoma	4 (5.7)	0 (0)	
T1b carcinoma	57 (81.4)	18 (100)	
SM depth, µm, median [range]	2000 [1200–4000]	2000 [1300–2625]	0.8845
Vascular invasion positive, *n* (%)	22 (31.4)	8 (44.4)	0.4035
Budding grade 2/3, *n* (%)	19 (27.1)	9 (50.0)	0.0885
En bloc resection, *n* (%)	70 (100)	18 (100)	–
VM distance, µm, median [range]	1000 [652–2025]	900 [500–1800]	0.5379
VM ≥500 µm, *n* (%)	56 (80.0)	16 (88.9)	0.5076
VM1, *n* (%)	0 (0)	0 (0)	–
Rebleeding, *n* (%)	2 (2.9)	1 (5.6)	0.5012
Perforation, *n* (%)	0 (0)	0 (0)	–

Abbreviations: EMR, endoscopic mucosal resection; ESD, endoscopic submucosal dissection; HGD, high‐grade dysplasia; muc, mucinous adenocarcinoma; pap, papillary adenocarcinoma; por, poorly differentiated adenocarcinoma; SM, submucosal; SD, standard deviation; sig, signet‐ring cell carcinoma; tub, tubular adenocarcinoma; VM, vertical margin.

**TABLE 5:** Clinicopathological characteristics and outcomes of tumors with the type B lifting condition in the endoscopic mucosal resection and hybrid endoscopic submucosal dissection groups.

	EMR group	Hybrid ESD group	
Variables	*n* = 23	*n* = 10	*p*‐value
Age, years, mean ± SD	69.8 ± 10.5	71.1 ± 10.4	0.7424
Sex, *n* (%)			
Male	15 (65.2)	5 (50.0)	0.4611
Female	8 (34.8)	5 (50.0)	
Location, *n* (%)			
Colon	19 (82.6)	6 (60.0)	0.2054
Rectum	4 (17.4)	4 (40.0)	
Tumor size, mm, mean ± SD	10.7 ± 4.5	15.2 ± 3.9	0.0108
Gross type, *n* (%)			
Protruded	16 (69.6)	5 (50.0)	0.4334
Superficial	7 (30.4)	5 (50.0)	
Main histology, *n* (%)			
tub/pap	21 (91.3)	10 (100)	1.0000
por/sig/muc	2 (8.7)	0 (0)	
Histology at the deepest invasive front, *n* (%)			
tub/pap	21 (91.3)	10 (100)	1.0000
por/sig/muc	2 (8.7)	0 (0)	
Histologic findings, *n* (%)			
HGD	1 (4.4)	2 (20.0)	0.2990
T1a carcinoma	1 (4.4)	0 (0)	
T1b carcinoma	21 (91.2)	8 (80.0)	
SM depth, µm, median [range]	2200 [1300–3000]	3000 [750–4125]	0.3446
Vascular invasion positive, *n* (%)	5 (21.7)	5 (50.0)	0.2148
Budding grade 2/3, *n* (%)	3 (13.0)	4 (40.0)	0.1605
En bloc resection, *n* (%)	21 (91.3)	10 (100)	1.0000
VM distance, µm, median [range]	0 [0–400]	700 [500–1925]	<0.0001
VM ≥500 µm, *n* (%)	2 (8.7)	9 (90.0)	<0.0001
Rebleeding, *n* (%)	0 (0)	0 (0)	–
Perforation, *n* (%)	0 (0)	0 (0)	–

Abbreviations: EMR, endoscopic mucosal resection; ESD, endoscopic submucosal dissection; HGD, high‐grade dysplasia; muc, mucinous adenocarcinoma; pap, papillary adenocarcinoma; por, poorly differentiated adenocarcinoma; SD, standard deviation; sig, signet‐ring cell carcinoma; SM, submucosal; tub, tubular adenocarcinoma; VM, vertical margin.

**TABLE 6:** Clinical characteristics of tumors with type A and B lifting conditions.

Variables	Type A *n* = 88	Type B *n* = 33	*p*‐value
Location, *n* (%)			
Colon	75 (85.2)	25 (75.8)	0.2809
Rectum	13 (14.8)	8 (24.2)	
Tumor size, mm, mean ± SD	12.2 ± 4.9	12.1 ± 4.8	0.8826
Gross type, *n* (%)			
Protruded	71 (80.7)	21 (63.6)	0.0591
Superficial	17 (19.3)	12 (36.4)	

Abbreviation: SD, standard deviation.

## DISCUSSION

Our study revealed indications for hybrid ESD for T1b CRCs ≤20 mm to ensure adequate VMs according to tumor lifting conditions. We recommend performing hybrid ESD in tumors with type B lifting conditions.

Given the increasing number of patients requiring T1 CRC treatment,^29^, ^30^, ^31^ the proportion of ER procedures for T1 CRC has increased.^1^ Because ER requires complete pathological resection for detailed pathological assessment, especially for T1 CRCs, a negative VM is an absolute requirement for curative resection.^2^, ^7^, ^8^ Additionally, as ER for T1b CRCs with VMs <500 µm or VM1 tumors affects patient prognosis,^8^ adequate VMs are important. We previously reported that submucosal fibrosis and poor differentiation at the deepest invasive portion are potential risk factors for VM1 in T1b CRCs,^32^ and that the EUS tumor‐free distance classification is a novel predictor of VM ≥500 µm before ESD.^33^ However, we did not confirm any appropriate ER method that ensures adequate VMs.

Colorectal hybrid ESD planned in advance is reportedly useful. A prospective, randomized controlled study in Korea showed that planned hybrid ESD for colorectal neoplasia ≥20 mm required a shorter time than did ESD (27.4 min vs. 40.6 min, *p* = 0.005), with similar en bloc resection (94.1% vs. 100%, *p* = 0.493) and perforation (8.8% vs. 6.5%, *p* > 0.999) rates.^21^ Okamoto et al.^34^ revealed that planned colorectal hybrid ESD is safe for lesions measuring ≥20–<30 mm and results in complete en bloc resection. However, no consensus regarding hybrid ESD indications has been reached, and its usefulness for cT1b CRC and lesions ≤20 mm remains unknown. In our study, we excluded CRCs treated using conventional ESD and simply compared EMR with hybrid ESD.

The non‐lifting sign,^16^, ^17^ which involves a simple yes/no classification, is a straightforward tool to determine EMR indications. Generally, normal saline or 10% glycerin solution is injected into the submucosa to assess the non‐lifting sign.^35^ For lesions with positive non‐lifting signs, ESD (including hybrid ESD) or surgical resection can be performed because EMR is generally difficult and en bloc resection is desirable.^24^ Because cT1b CRCs resulting in VM1 with a negative non‐lifting sign after ER are not rare, a more detailed classification is necessary. Kato et al.^36^ assessed the indications for and limitations of EMR for early CRC, focusing on tumor lifting after submucosal injection, and reported that these indications and limitations were easily assessed based on the tumor's lifting characteristics, which were significantly related to the invasion depth. We selected the tumor lifting condition after submucosal injection as a clinical tumor characteristic and classified it as type A, type B, or non‐lifting. Non‐lifting is characterized by a positive non‐lifting sign.

Based on our results, type B tumors were associated with VM1 and VM <500 µm. Type A tumors showed no significant between‐group differences, in contrast, for type B tumors, the VM distance was significantly longer and the proportion of VMs ≥500 µm was significantly higher in the hybrid ESD group than in the EMR group. Therefore, we suggest the following strategy for cT1b CRCs ≤20 mm to ensure adequate VMs. When the tumor lifting condition is type A, EMR can be performed; however, hybrid ESD can be considered for type A tumors that are difficult to resect with snaring alone because of their location, and for tumors that are difficult to classify as type A or B. When the tumor lifting condition is type B, hybrid ESD can be performed. Because the sample size for non‐lifting tumors was very small, we could not determine whether ESD (including hybrid ESD) or surgical resection is appropriate for non‐lifting tumors. Moreover, we could not identify any pre‐injection predictors of type B lifting conditions or specific differences in preoperative EUS findings between the lifting conditions.

Endoscopic images of EMR procedures for tumors with type B lifting conditions revealed that the snare tended to slip on the lifted mucosa during snaring and a smaller margin could be obtained, possibly resulting in a shorter VM distance. This can frequently occur in tumors with non‐lifting conditions. For lesions with massive submucosal invasion, dense fibrosis associated with invasive carcinoma prevents fluid infiltration through the submucosal connective tissue, thereby preventing bleb formation beneath the lesion, and the lesion is not elevated (non‐lifting sign).^17^ Therefore, the type B lifting condition is expected to be a prelude to the non‐lifting sign.

This study had some limitations. First, as a single‐center retrospective study, it had some inherent biases. Additionally, conventional ESD was excluded; these cases may show deeper invasion, and this could indicate a selection bias for cT1b CRCs. We performed preoperative EUS for all cT1b CRCs at our institution, and conventional and hybrid ESD was performed after confirming a certain distance between the deepest invasive tumor front and muscle layer. Second, indications for EMR and hybrid ESD were determined at the individual operator's discretion. Third, the sample size was relatively small, with few opportunities to select ER instead of surgical resection for cT1b CRCs. Fourth, the submucosal injection position is around the lesion in hybrid ESD and under the lesion in EMR; this slight difference may have partially affected decisions and tumor‐lifting conditions. However, this study targeted relatively small lesions (<20 mm); therefore, even during hybrid ESD, the first local injection is performed from the lesion's anal side to just below the lesion, and we evaluated the tumor lifting conditions during the first local injection. Finally, although the operators attempted to perform dissection just above the muscle layer before snaring during hybrid ESD, the treatment technique undeniably affected the results.

In conclusion, our novel classification of tumor lifting conditions can be a useful indicator for guiding decisions regarding the selection of EMR or hybrid ESD to ensure adequate VMs for cT1b CRCs ≤20 mm.

## CONFLICT OF INTEREST STATEMENT

None.

## ETHICS STATEMENT

This study was conducted in accordance with the Declaration of Helsinki. The use of patient data was approved by the Institutional Review Board of Hiroshima University (registration number: E‐241).

## PATIENT CONSENT STATEMENT

N/A.
